# CD7 Positive Diffuse Large B-Cell Lymphoma Arising in a Background of Follicular Lymphoma: A Case Report and Review of the Literature

**DOI:** 10.1155/2016/5415974

**Published:** 2016-09-28

**Authors:** Elham Vali Betts, Hooman H. Rashidi

**Affiliations:** Department of Pathology and Laboratory Medicine, University of California, Davis, 4400 V Street, Sacramento, CA 95817, USA

## Abstract

Diffuse large B-cell lymphoma (DLBCL) is a neoplasm of large B-lymphocytes with a diffuse growth pattern. The neoplastic cells express B-cell markers such as CD20 and PAX-5 and there may be coexpression of BCL-2, BCL-6, CD10, and MUM-1. With the exception of CD5, other T-cell markers are not commonly expressed in this neoplasm. Here, we describe the first reported case of a DLBCL with abnormal expression CD7 arising in a background of follicular lymphoma in an 81-year-old male who presented with a nontender left axillary mass. Additionally, no other T-cell antigens were expressed in this B-cell lymphoma. Expression of CD7 in DLBCL is exceptionally rare and its prognostic significance is unknown. Here, we describe this rare case with review of literature of known DLBCLs with expression of T-cell antigens.

## 1. Introduction

According to WHO 2008 diffuse large B-cell lymphoma (DLBCL) is a neoplasm of large B-cells with nuclear size equal to or exceeding normal macrophage nuclei or more than twice the size of a normal lymphocyte that has a diffuse growth pattern and can arise de novo or as a result of progression or transformation of a low grade B-cell lymphoma. The neoplastic cells in DLBCL typically express pan B-cell markers such as CD20, CD19, CD79a, and PAX-5. There may be coexpression of CD10, BCL-6, BCL-2, CD30, and MUM-1 and in some cases they may express CD5. However, expression of non-CD5 T-cell antigens (CD2, CD3, and CD7) in DLBCL is extremely rare [1].

## 2. Case Presentation

**Figure 1:**
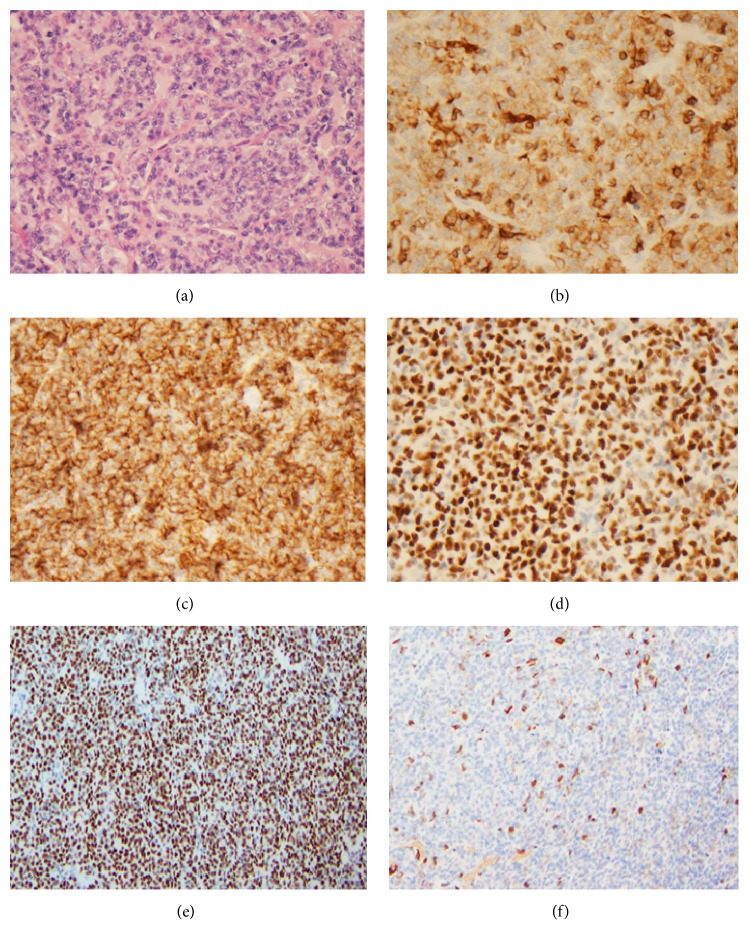
(a) H&E section of the diffuse area. (b) CD7 expression in diffuse areas. (c) CD20 expression in diffuse areas. (d) BCL-6 expression in diffuse areas. (e) BCL-6 expression in follicular area. (f) CD7 negative in follicular lymphoma area.

**Figure 2:**
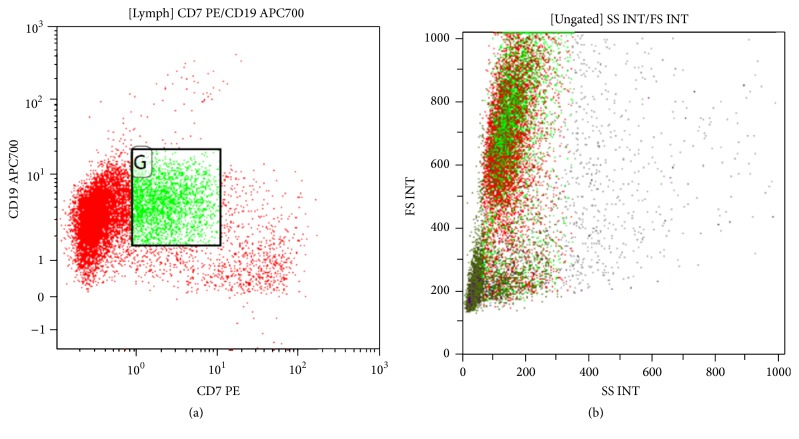
Neoplastic cells express CD19 with a subset with expression of CD7. These cells have a high forward scatter, consistent with the CD7 positive large cells.

An 81-year-old male with a history of prostate cancer presented with malaise and myalgias of several months. Subsequently, a left axillary mass was discovered and an excisional biopsy was performed. Microscopic examination of the mass revealed a follicular pattern with large atypical cells in one section and areas of diffuse proliferation of large atypical cells with irregular, large nuclei with vesicular chromatin and prominent nucleoli in other sections. By immunohistochemistry the neoplastic cells in the diffuse area were positive for B-cell markers (CD20 and PAX-5) and negative for the T-cell marker CD3 (Figure 1). These cells coexpressed BCL-2, BCL-6 (Figure 1), and a small subset express MUM-1 and were negative for BCL-1. Interestingly, flow cytometry showed the coexpression of CD7 in a subset of the monotypic B-cells (Figure 2). Follow-up immunohistochemistry also showed CD7 expression but this coexpression was confined to the large cells (specifically the areas involved by the diffuse large B-cell lymphoma) (Figure 1). Notably the areas involved by the follicular lymphoma were negative for CD7 (Figure 1). The histologic and immunophenotypic findings are consistent with a CD7 positive diffuse large B-cell lymphoma arising in a background of a CD7 negative follicular lymphoma grade 3B. An IgH molecular analysis was not performed. Pelvic, abdomen, and chest CT showed left axillary lymphadenopathy and an enlarged hilar lymph node. Bone marrow biopsy did not show any involvement by lymphoma.

## 3. Discussion

This case is extremely rare and highlights the abnormal expression of the T-cell marker, CD7, in a diffuse large B-cell lymphoma arising in in a background of CD7 negative follicular lymphoma. CD7 is a membrane bound glycoprotein [2], a member of the immunoglobulin superfamily [3], and is the first T-cell lineage-associated antigen expressed in T-cells [4]. CD7 is a 40 kDa polypeptide that acts as costimulator for tyrosine and lipid kinase activity [3]. The exact role of CD7 is unknown [5]. Only very rare cases of diffuse large B-cell lymphoma with aberrant expression of non-CD5 T-cell markers such as CD2 and CD7 have been reported (Table 1), none of which to our knowledge have CD7 expression while arising in a background of a CD7 negative follicular lymphoma (Figure 1). In one study Inaba et al. described 10 cases of DLBCL of which 3 cases expressed CD7 [6] and in another study Inaba and colleagues evaluated 4 cases of DLCBL of which none of them showed expression of CD7 [7]. The significance of these aberrancies is unclear, but it is important to be aware of such findings since it may lead to diagnostic dilemmas. Expression of T-cell markers based on previous studies has not been associated with an aggressive clinical course [8]. Further studies are also important to elucidate the prognostic impact of such markers in DLBCLs.

## Figures and Tables

**Table 1:** Immunohistochemical expression of non-CD5 T-cell antigens in DLBCLs. The manuscripts noted in the table do not discriminate the various non-CD5 positive DLBCLs in germinal center versus nongerminal center phenotype. According to Suzuki et al., the rate of non-CD5 T-cell antigen expression in DLBCLs is extremely low with only ~0.3% of their known cases. Inaba et al. evaluated 10 cases of DLBCL of which 3 of them showed expression of CDC7 [6]. In another study Inaba et al. showed no cases of DLBCL with expression of CD7 by evaluation of 4 cases [7].

Number	Authors	Total patients with DLBCL	De novo/transformed	CD2	CD3	CD4	CD5	CD7	CD8	Method
1	Suzuki et al. [9]	150	De novo	1	0	0	ND^*∗∗∗*^	3	1	IHC^*∗*^
2	Inaba et al. [6]	10	De novo	1	0	0	6	3	0	FC^*∗∗*^
3	Kaleem et al. [8]	2	De novo	1	0	1	0	1	0	FC
4	Inaba et al. [7]	4	De novo	0	0	0	4	0	0	FC
5	E. Vali Betts	1	Transformed	0	0	0	0	1	0	FC and IHC

^*∗*^Immunohistochemistry.

^*∗∗*^Flow cytometry.

^*∗∗∗*^Not done.
